# Investigation of immunogenic properties of Hemolin from silkworm, *Bombyx mori* as carrier protein: an immunoinformatic approach

**DOI:** 10.1038/s41598-018-25374-z

**Published:** 2018-05-03

**Authors:** Veeranarayanan Surya Aathmanathan, Nattarsingam Jothi, Vijay Kumar Prajapati, Muthukalingan Krishnan

**Affiliations:** 1 Department of Environmental Biotechnology, Bharathidasan University, Tiruchirappalli, Tamil Nadu India; 2 Department of Biochemistry, School of Life Sciences, Central University of Rajasthan, Bandarsindri, Kishangarh, Ajmer, 305817 Rajasthan India

## Abstract

Infectious diseases are the major cause of high mortality among infants and geriatric patients. Vaccines are the only weapon in our arsenal to defend us ourselves against innumerable infectious diseases. Though myriad of vaccines are available, still countless people die due to microbial infections. Subunit vaccine is an effective strategy of vaccine development, combining a highly immunogenic carrier protein with highly antigenic but non–immunogenic antigen (haptens). In this study we have made an attempt to utilize the immunoinformatic tool for carrier protein development. Immunogenic mediators (T-cell, B-cell, IFN-γ epitopes) and physiochemical properties of hemolin protein of silkworm, *Bombyx mori* were studied. Hemolin was found to be non-allergic and highly antigenic in nature. The refined tertiary structure of modelled hemolin was docked against TLR3 and TLR4-MD2 complex. Molecular dynamics study emphasized the stable microscopic interaction between hemolin and TLRs. *In-silico* cloning and codon optimization was carried out for effective expression of hemolin in *E. coli* expression system. The overall presence of Cytotoxic T Lymphocytes (CTL), Humoral T Lymphocytes (HTL), and IFN-γ epitopes with high antigenicity depicts the potential of hemolin as a good candidate for carrier protein.

## Introduction

Encapsulated bacteria are important pathogens that cause disease especially among infants, elderly and immunocompromised persons. Almost all encapsulated bacterial pathogens cause disease in children involves virulence factors such as surface capsular polysaccharides^1^. Polysaccharides are less immunogenic and it protect from protease activity of the host. The polysaccharide vaccines were poorly immunogenic and did not induce protective immunity in children younger than 18 months^2^. In the notion to overcome the above mentioned difficulties, larger proteins with high immunogenic properties are conjugated with the small antigenic molecules (haptens) to elicit a stronger immuongenic response with prolonged T-cell memory^3^. Hemolin a 48 kDa protein which is present in major lepidopteran insects, that is similar to immunoglobulin which consists of four immunoglobulin-like domains of the C2 type^4,5^. It exhibit both antiviral and antibacterial activities. It is mainly synthesized in the fat body and gut of the insects^6,7^. Hemolin transcript is not only induced by bacteria, but also by bacterial components like lipopolysaccharide and phorbol 12-myristate 13-acetate^8^. Functional analysis of expressed hemolin revealed that it binds to the components of bacterial cell wall and form a complex with other two hemolymph proteins^9^. The antimicrobial activity of hemolin is brought forth by the involvement of hemocytes. The activity of hemolin is related to the function of Toll like Receptor (TLR’s) in humans which confers to the activation of innate immunity in humans^10^. Some reports suggested that hemolin is highly expressed when it is challenged with dsRNA or Baculovirus^11^. Hemolin shares 28% sequence similarity with the vertebrate axon surface protein, axonin 1, expressed in the developing retina of humans^12^. The presence of cell adhesion motifs in hemolin, elicits a stronger interaction between antigen and protein. The intron positioning both within and between the immunoglobulin-like domains, further proves the feature which is typical for cell-adhesion molecules belonging to the immunoglobulin superfamily like the (Neural cell adhesion molecule) NCAM’s^13^. These adhesion properties of hemolin make it a suitable candidate for carrier protein in the development of vaccines for small antigenic molecule (haptens). The large size of Hemolin and distant phylogenetic relation with humans makes hemolin a highly immunogenic antigen. Combined adhesion property and high immunogenicity of hemolin makes it a promising contender for carrier protein in vaccine development. One current hypothesis is that hemolin might be a ‘pattern recognition receptor’, which discriminates between self and infectious non-self by the recognition of molecules unique to microorganisms, for example, Lipopolysaccharide (LPS)^14^. Based on this, the present hypothesis is framed to develop Hemolin-antigen conjugate to elicit a better T-cell response and herd immunity.

The major TLRs involved in bacterial infection are TLR-2 and TLR-4. Lipopolysaccharides are endotoxins specifically recognized by heterodimer receptor of TLR4-MD2 complex^15^. Lipopolysaccharides are transferred to the heterodimer complex with the help of LPS- binding protein (LBP) and CD4. Recognition of gram negative bacteria by TLR4 involves a cascade of interactions. The LPS is first bound by the LBP and transferred to CD14, in the next step the CD14 transfers the LPS to the TLR4-MD2 complex. The CD14 can also bind other bacterial components like lipoproteins, lipoteichoic acid (LTA), or lipoglycans. The lipid A domain which is highly conserved and consists of long acyl chains in LPS is specifically recognized by the MD2 complex^16^. Deletion mutation of TLR4 did not recognize LPS, rather with MD2 complex the function of TLR4 was restored. TLR4 and MD2 acts in a synergistic manner to recognize the bacterial LPS^17^. TLR-3 is specifically involved in viral infections. The location of TLR3 is in endoplasmic reticulum of the uninfected cells. The viral infection stimulates the translocation of TLR3 from endoplasmic reticulum to the cell surface^18^. The activation of TLR3 is a pH dependent dimerization process involving membrane protein UNC-93B. TLR3 mainly recognizes viral dsRNAs with 45 bp in length, the size of the viral dsRNA is crucial for signaling. The dsRNA triggers TRIF recruitment by TLR3 dimerization and Tyr phosphorylation, activating the cascade of signaling molecules IRF3, NF- κB and AP-1 which arms the sentinels against the viral infection^19^.

Hemolin acts as a pattern recognition molecule in silkworm, *Bombyx mori* and activates opsonisation and melanization in silkworm upon microbial infections. Hemolin also have affinity towards both bacterial and viral cell components^6^. Recent studies have proved that TLR activation plays a major role in vaccination memory and herd immunity. Here, first time, we have investigated the immunogenic properties of hemolin protein using several immunoinformatics approaches^20,21^. In this study we docked the Hemolin protein with TLR3 and TLR4-MD2 complex to study the interaction of hemolin with the TLRs. We have also used B cell and T cell prediction tools to analyze the antigenic properties of hemolin. The stability of the TLR–hemolin complex was studied using molecular dynamics study using GROMACS.

## Results and Discussion

### Collection of protein sequences and PDB structures

The hemolin sequence of silkworm, *Bombyx mori* was retrieved as FASTA format from UNIPROT database. The SMART analysis tool revealed four Ig2 like domains in hemolin protein (Supplementary Fig. 1). The values of the IG2 domain given by SMART tool analysis were highly significant, which validated the immunoglobulin like domains in Hemolin. The PDB structures of TLR3 and TLR4-MD2 complex were retrieved from RCSB Protein Data Bank. The TLRs retrieved from PDB was used for docking studies. The TLRs membrane proteins, majorly present in dendritic cells and are activated upon microbial infections.

### Cytotoxic T Lymphocyte (CTL) epitope prediction Helper T Lymphocytes (HTL) epitope prediction of hemolin

The Helper T Lymphocytes (HTL) and Cytotoxic T Lymphocytes (CTL) plays an important role in mounting adaptive immune response against various microbial infections. The MHC class I and class II recognition plays an important role in extracellular and intracellular infections^22^. The cytotoxic T lymphocyte epitope predictions were carried out using NETCTL 1.2 server. The NETCTL server use an algorithm which takes combined scores of peptide proteasomal C terminal cleavage, MHC class I binding, and TAP transport efficiency for epitope prediction. The epitopes were selected which had threshold values greater than 1.25, thereby increasing the specificity of peptide towards MHC class I molecules. A total of 34 CTL epitopes (9 mers) were identified to be recognized by MHC class 1 molecules (Supplementary Table 1). The HLAs (Human Leukocyte Antigen) play a major role in recognizing the antigen presented by the TLRs. The Helper T cells upon activation sets a series of chain reaction activating macrophages, cytotoxic T Lymphocytes and B- cell mediated immune response resulting in full-fledged immune response against the invading pathogen^23^. The 15mer HTL epitopes of hemolin protein was predicted using IEDB tools. The results were given as percentile rank. The lowest the percentile rank has the highest IC50 value. The IEDB server gave ten peptides for the hemolin protein, percentile ranks ranging from 0.99–2.50 (Table 1). The low percentile ranks of peptide shows that, the hemolin protein has good antigenic property and is capable of eliciting Helper T cell response. The combined results of CTL and HTL epitope prediction proves the capability of Hemolin protein to be recognized by MHC class I and II molecules.

**Table 1:** HTL epitope prediction by IEDB Server.

Allele	Start	End	Sequence	Percentile rank
H2-IAd	286	300	SGRRLVIKEVWAEDA	0.99
H2-IAd	287	301	GRRLVIKEVWAEDAG	1.04
H2-IAd	193	207	DKTKLVCMASSPAAD	1.71
H2-IAb	213	227	VTYYITQVTPASEPT	1.82
H2-IAb	212	226	IVTYYITQVTPASEP	1.85
H2-IAd	192	206	NDKTKLVCMASSPAA	2.08
H2-IAd	396	410	NEHGAEYAETALQVA	2.34
H2-IAb	211	225	PIVTYYITQVTPASE	2.35
H2-IAb	214	228	TYYITQVTPASEPTY	2.51
H2-IAb	210	224	VPIVTYYITQVTPAS	2.58

### B-cell epitope prediction of Hemolin

**Figure 1:**
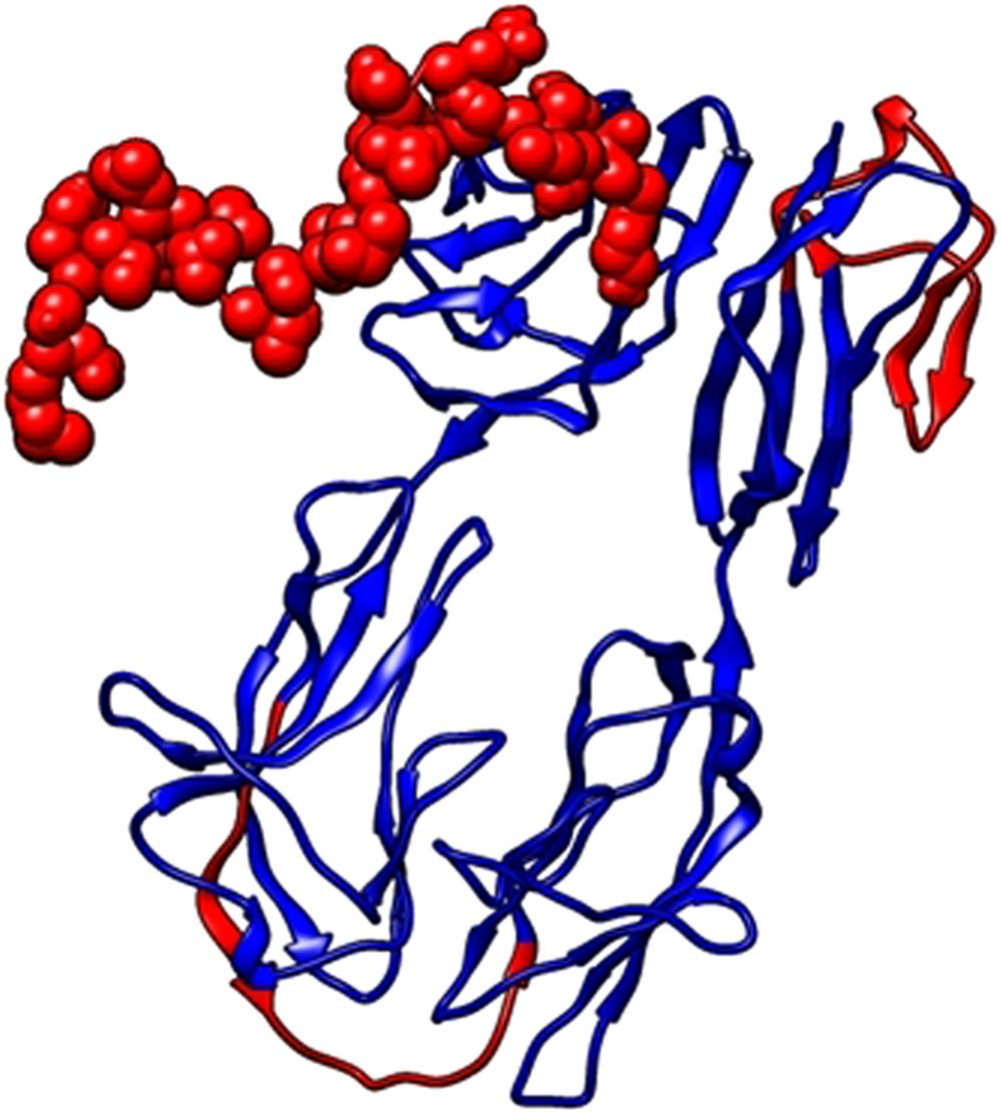
B- cell epitope prediction, discontinuous B- cell epitope have been represented as red spheres in carrier protein and linear epitopes are represented as red lines.

The B-cells constitutes the major arm of humoral immune response. The activation of B-cells by the helper T cells will increase differentiation of B-cells and triggers an immune response. The carrier protein must be also identified by the B-cells to increase the immunoglobulin levels to attain good primary and secondary immune response^22^. The B cell epitope was predicted using BCPREDS server. Total of 11 epitopes of 20mers were identified to be recognized by B-cells, the epitopes were selected with high scores (Table 2). The B-cell epitopes with high scores tends to validate the high immunogenic property of the Hemolin protein. The Ellipro suite was used to identify the linear and discontinuous epitopes. The epitopes were identified based on Thornton’s method and it used MODELLER structure prediction tools and Jmol, a java based web applet for 3D visualization of predicted epitopes. Total of 99 conformational residues were identified as both continuous and discontinuous epitopes. There were three discontinous epitopes with high scores (MET1 to LYS26, THR214 to ILE 232 and TYR363 to TYR390) and one linear epitope with high scores ranging from (MET1 to LYS26). The residue scores of starting MET1 was 0.985 and LYS26 was 0.471. (Figure 1) (Supplementary Table 2).

**Table 2:** B- cell epitope prediction by BCPREDS Server.

Position	Epitope	Score
11	TCVIYTTGQPVNSGKVPVL	0.998
138	FELRCPVPGGYPKPTISWMR	0.995
48	ECATEGDDSGVEYSWRKDGM	0.985
201	ASSPAADEGVEYSWRKDGM	0.982
384	KSNQGYYGCTASNEHGAEYA	0.975
323	VVSAPTFTTKPEKRTLATQG	0.969
269	VNVDNTYKDRITRHNRSSGR	0.961
159	HDEDGSTENFMDRRATYSPE	0.961
344	DVTIPCKATGIPSPLVSWTY	0.957
294	EVWAEDAGTYTCDVDNQAGR	0.925
87	TQTKASDEGEYQCFAKSDFG	0.806

### Prediction of allergenicity and physiochemical parameter of hemolin

The allergenicity was predicted using allerdictor fast allergen prediction tool using SVM method. Hemolin was classified as non-allergic with a score of −0.983, whereas the probable allergenicity score was 0.006. The physiochemical parameters of hemolin was studied using Protparam server. The molecular weight of the protein was found to be 48 kDa, the high molecular weight emphasizes the antigenic nature of hemolin. Theoretical isoelectric point of hemolin was found to be pI 5.12, which makes the protein net charge to be negative. These physiochemical parameter facilitates us during the chromatographic separation of the protein for *in vivo* studies. The extinction coefficient was found to be 62800 at absorption of 0.1% proving that all cysteine residues are reduced. The half-life analysis of the hemolin was determined as 30 hours in mammalian reticulocytes (*in vitro*), 20 hours in yeast (*in vivo*) and 10 hours in *E. coli* (*in vivo*). The stability score was found to be 38.90, which indicates that the protein is highly stable. The aliphatic index was 68.46 shows the protein has many hydrophilic residues. Grand average of hydropathicity (GRAVY) score was −0.433, negative GRAVY score indicates that the protein is non-polar.

### Interferon γ (IFN- γ) inducing epitope prediction and Antigenicity prediction of hemolin

**Figure 2:**
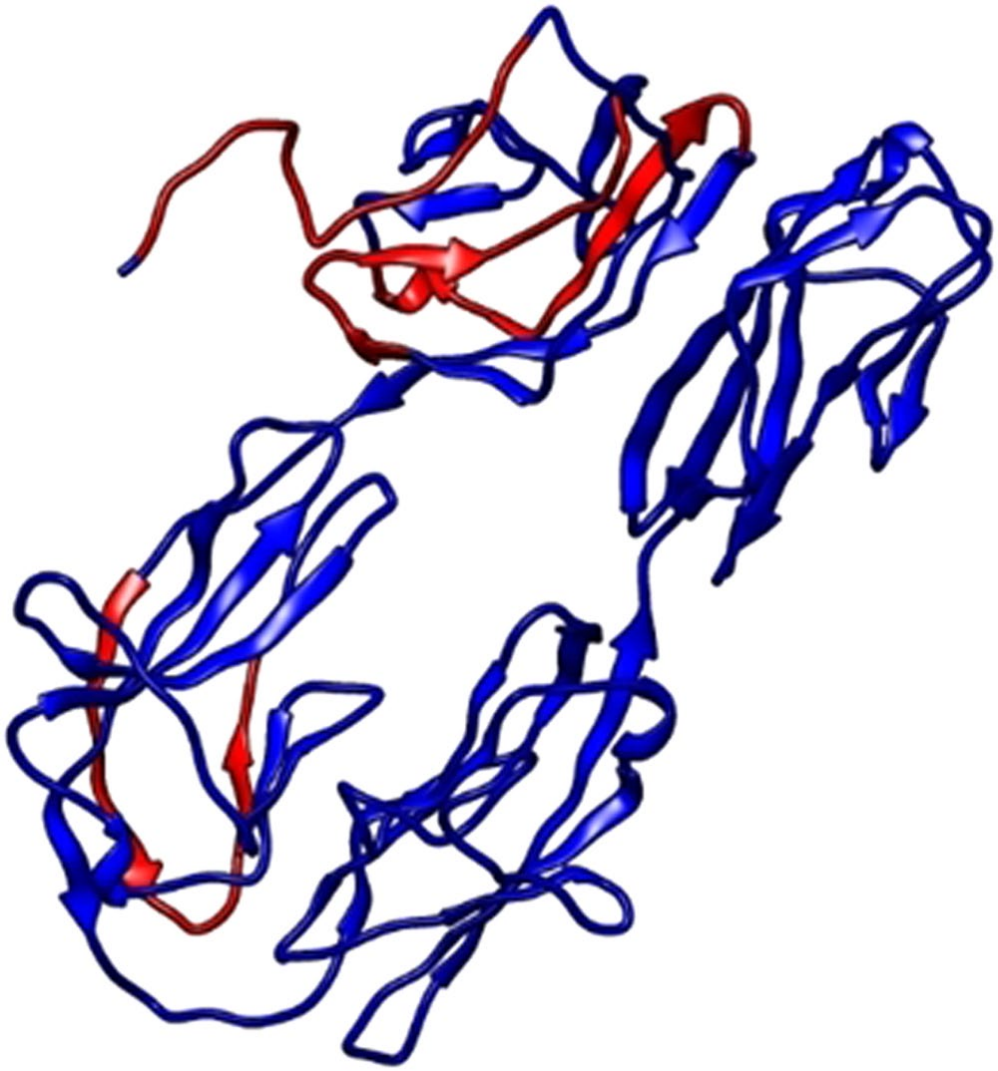
IFN γ epitope prediction, linear IFN γ epitope have been shown in red color in carrier protein.

The IFN **γ** plays an important role in intracellular pathogen evasion and majorly acts as cytokines for natural killer cells and cytotoxic T lymphocytes. The 15 mer IFN **γ** specific epitopes were predicted using MERCI based motif search. Total of 402 epitopes were identified, out of which epitopes with both positive and negative scores were present including 71 IFN **γ** epitopes. The epitopes with scores greater than threshold 0.4 was highlighted in Fig. 2. The antigenicity of Hemolin was predicted using ANTIGEN Pro and VaxiJen servers. The antigenic property of the carrier protein solely decides the effectiveness of the vaccine. The antigen might be non-immunogenic but it’s very important for the carrier protein to be highly immunogenic in order to mount a strong immune response. The highly antigenic vaccine are more liable to trigger stronger and long lasting T- cell memory and B-cell immune response. ANTIGEN Pro server predicted the probability of antigenicity score to be 0.9228. The VaxiJen server predicated an antigenicity score of 0.6234 for hemolin, which was above the default threshold value which was 0.5. Both antigen and IFN **γ** prediction delivered a higher antigenicity score than the threshold, proving hemolin to be a strong immunogenic, which could possibly accomplish the task of stronger and long lasting immune response.

### Secondary and tertiary structure prediction of hemolin

**Figure 3:**
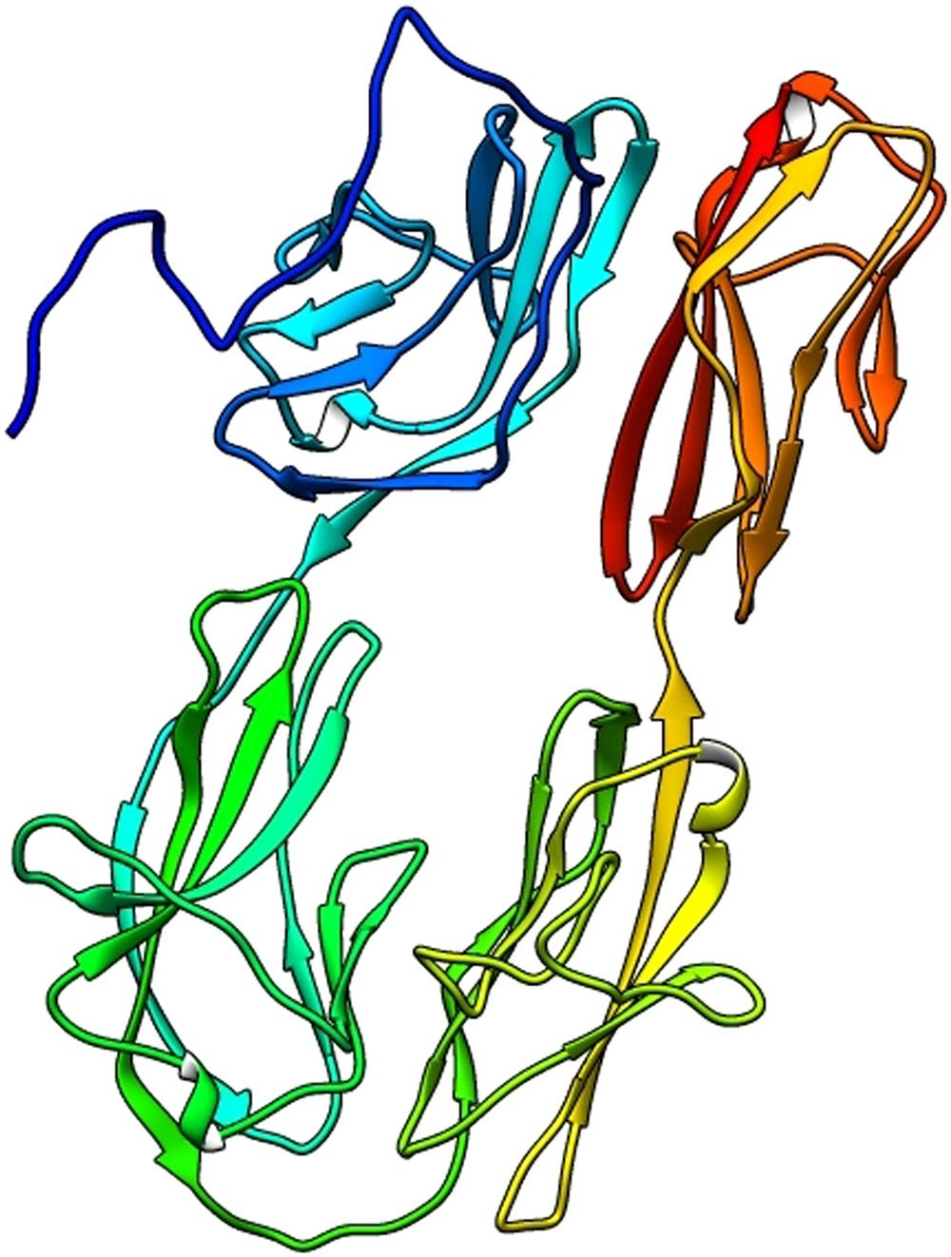
Final 3D model of hemolin protein after homology modeling and refinement.

The secondary structure prediction using PSIPRED server for Hemolin revealed the existence of 58% β sheet and 41% coils. The β sheets and coils are the classic structure of Ig like domains which validates the SMART tool analysis result. This result correlate with the finding of Bettencourt *et al*.^12^. The tertiary structure of hemolin was predicted using RaptorX, the structural prediction revealed that hemolin has single domain which was construted based on the best fitting template (PDB ID: 1BIH). A total of 410 aa was modelled with 6% disorderness (Fig. 3). The p-value of the homology modelled structure inidcates the quality of the modelled 3D structure. A P-value less than 10^−4^ is a good for proteins with predominantly with β sheets. The P-value of the predicted hemolin was found to be 6.87e–18, which is less the threshold p-value that strongly validates the stability and quality of the modelled protein.

### Tertiary structure refinement and validation

**Figure 4:**
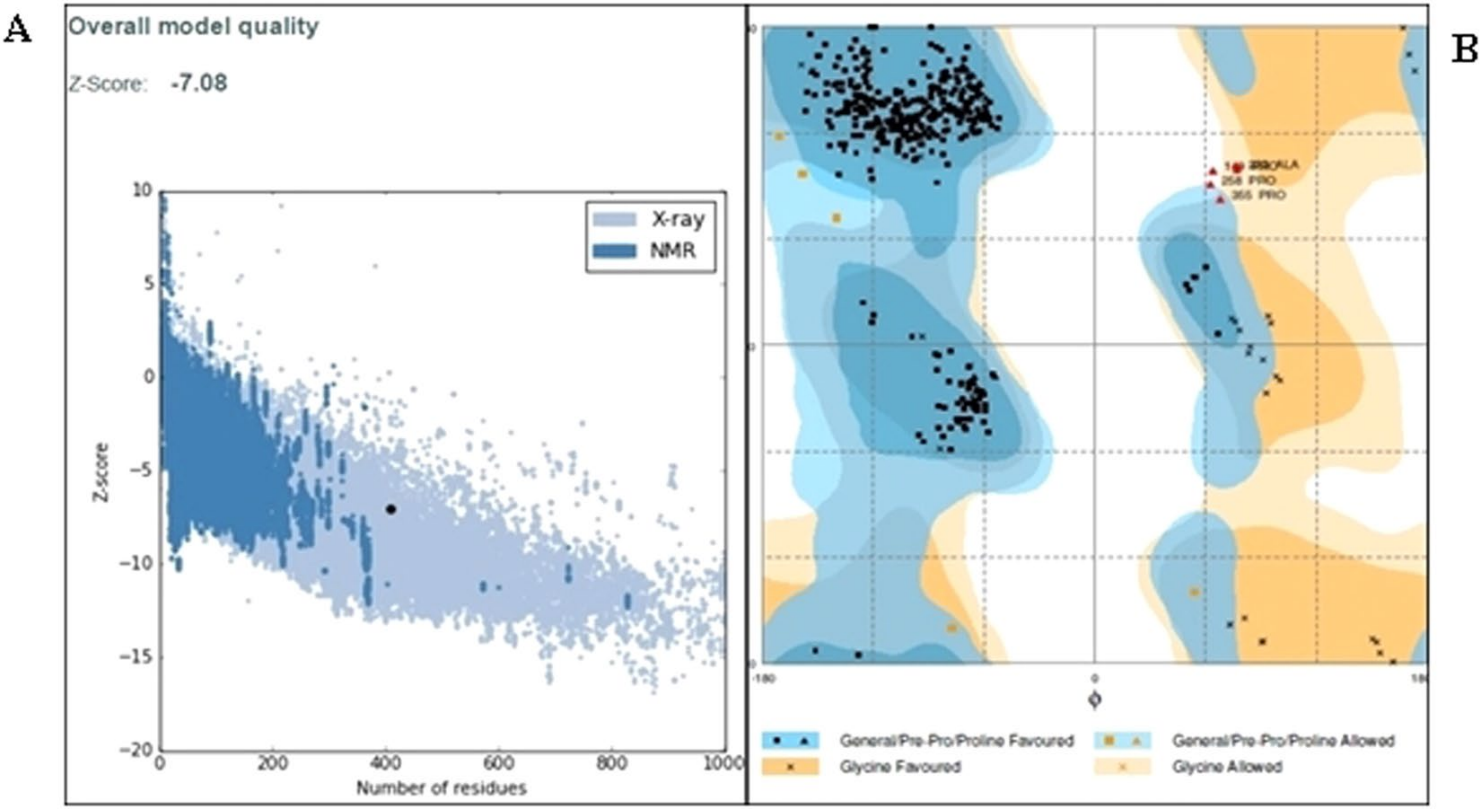
Validation of 3D hemolin protein model. (**A**) PROSA validation showing Z- Score (−7.08) and (**B**). Ramachandran plot analysis of structure showing 97.8%, 1.2% and 1.0%residues in favored, allowed and disallowed region.

The quality of the modelled protein was enhanced using the Galaxy refine server. The loop refinement and energy minimization was carried out to achieve maximum quality of the predicted model. Galaxy refine server generated 5 models, out of which one exemplary model with GDT-HA (0.9585), RMSD (0.397), Molprobity (1.686), clash score (12), poor rotamers (0.3), Rama favored region (97.5%) was selected for further analysis. The Ramachandran plot for the refined structure was plotted using the RAMPAGE server, plot revealed a 98% of residues in favored regions and 2% in allowed regions (Fig. 4B). The potential errors and quality of analyzed using ERRAT and ProsaWeb servers. The overall quality factor of modelled hemolinprotein was 88.9% as predicted by ERRAT server. The Z score for the hemolin protein was predicted to be −7.08 by ProsaWeb server (Fig. 4A), the high negative score ensures the maximum quality of the modelled hemolin protein. The overall results from RAMPAGE, ERRAT and Prosaweb has validated the high quality of the 3D modelled hemolin protein.

### Molecular docking of hemolin with TLR 3 and TLR-MD2 complex

**Figure 5:**
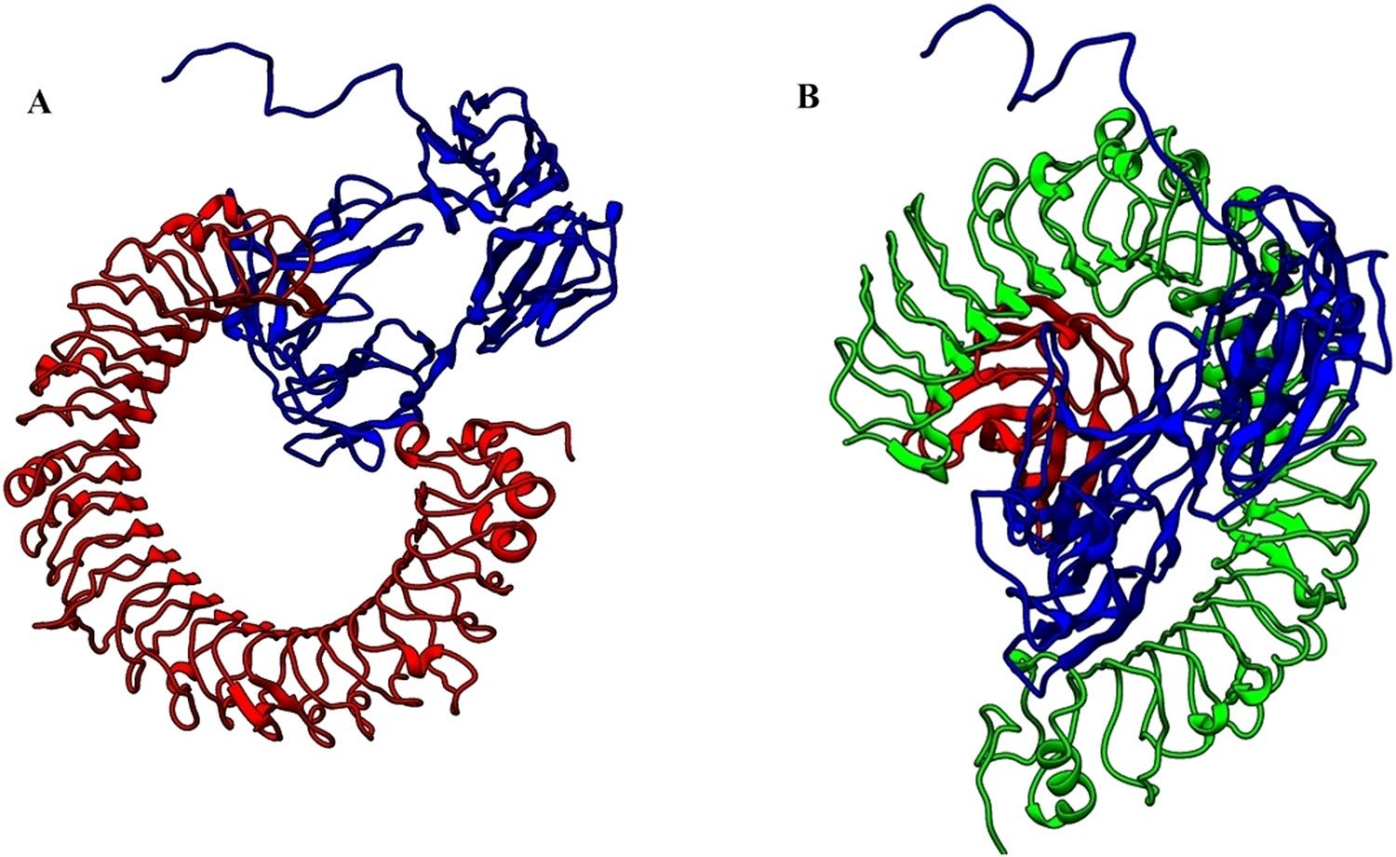
Molecular docking by patchdock server. (**A**) TLR- 3 represented in red color and hemolin protein in blue color, (**B**). Complex from patchdock server showing TLR – 4 in green color, MD2 in red color and hemolin protein in blue color.

The interaction between the TLRs and hemolin was evaluated using the PatchDock server. The PatchDock protein-protein docking algorithm calculates the surface binding affinity of the receptor and ligand proteins using object recognition and image segmentation analysis. The hemolinwas were docked against TLR3 and TLR4-MD2 complex separately and the results were analyzed. The docked results were refined using FireDock server based on the global energy of the docked complex. The FireDock server generated ten best models with least global energy confirming best affinity between the TLRs and hemolin protein. The docking score was found to be −11.30 and −20.37 for complexes Hemolin-TLR3 (Fig. 5A) and Hemolin-TLR4-MD2 complex (Fig. 5B), respectively. The docked complexes were further taken for molecular dynamic analysis.

### Molecular dynamics simulation of receptor-ligand complex

**Figure 6:**
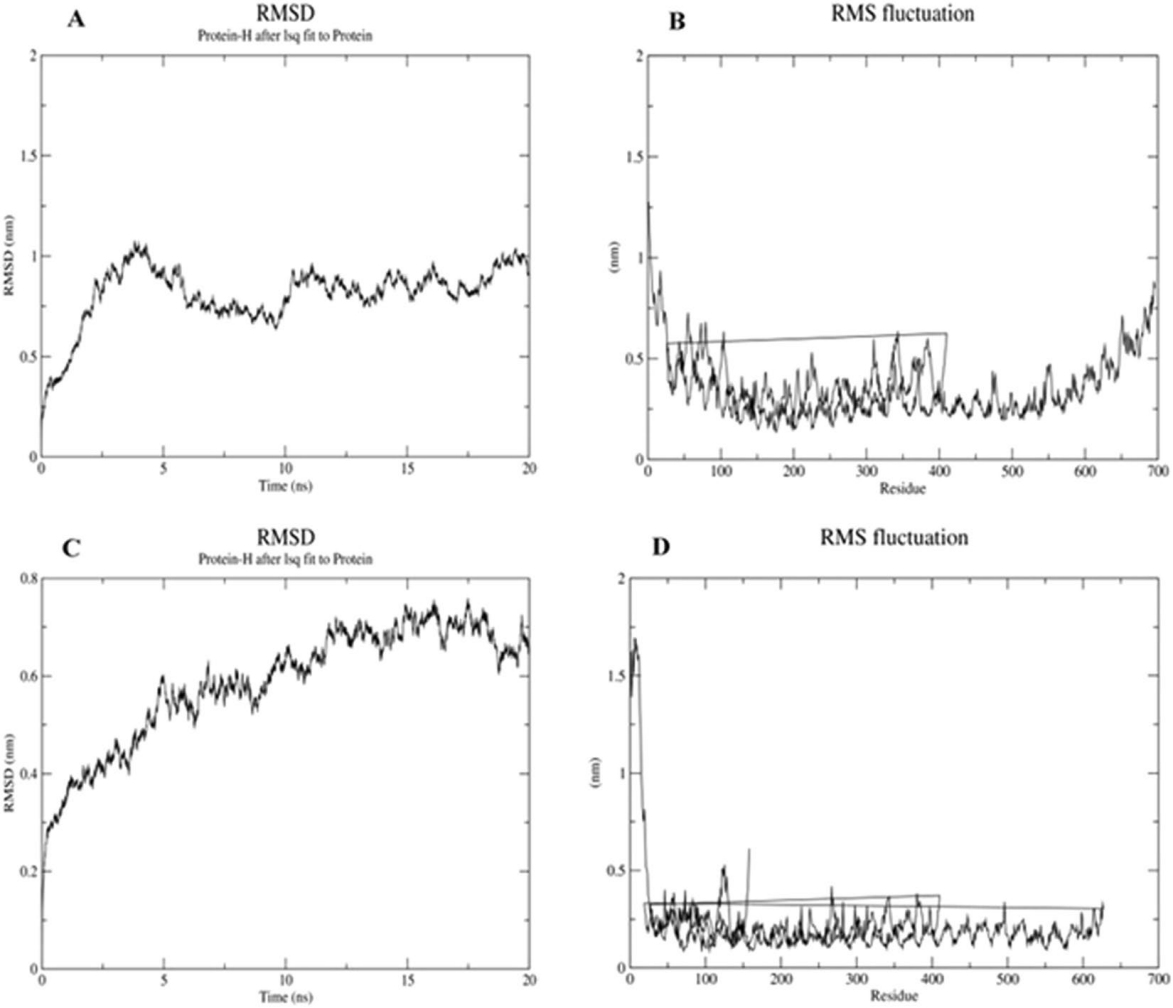
Molecular dynamics simulation study of Protein – protein complex for the timeduration of 20 ns. (**A**) RMSD of docked complex hemolin with TLR -3, (**B**). RMSF docked complex hemolin with TLR -3, (**C**). RMSD of hemolin and TLR- 4 MD2 docked complex, (**D**) RMSF of hemolin and TLR- 4 MD2 docked complex.

Molecular dynamics analysis helps us to study the stability of docked complexes at varying thermo baric conditions. The docked complexes were subjected to molecular dynamics study using GROMACS v 5.1.4 software. The software was used to check the stability and interaction between hemolin and TLRs (TLR3 and TLR4-MD2 complex) docked complexes at microscopic levels. The energy components, potential energy, density, pressure, temperature and volume calculations for the docked complexes were studied using varying parameters. The temperature was maintained at 300 K for 100 ps interval and the pressure was maintained at 1 bar for the same time interval, minimal fluctuations were observed during the 100 ps time span for both Hemolin- TLR3 and Hemolin-TLR4-MD2 complexes. The structural stability and interaction of the docked complexes were observed by studying the root mean square deviation (RMSD). The RMSD was calculated by measuring the distance between the backbone conformation of ligand and receptor protein. The RMSD value of Hemolin-TLR3 complex started to fluctuate at 0.2 nm and continued for a time span of 20 ns ending at 0.9 nm (Fig. 6A), showing stability for a very long duration. The root mean square fluctuation (RMSF) for the same complex started to fluctuate at 1.25 nm in the initial residues, residues at the middle of the complex fluctuated at average of 0.5 nm and again the fluctuation of final residues increased and ended at 0.9 nm (Fig. 6B). The RMSF value indicates the presence of flexible regions are present at both ends of the docked complex. The RMSD of Hemolin – TLR4-MD2 complex started to fluctuate at 0.2 nm and continued for 20 ns and ended at 0.65 nm (Fig. 6C) showing higher structural stability and strong interaction between the hemolin and TLR4-MD2 complex. The RMSF fluctuation was observed at 1.5 nm at initial residues and it decreased below 0.5 nm till the last residue in the complex (Fig. 6D). The RMSD results show us the stronger affinity of hemolin with TLR4-MD2 complex than TLR3 complex. This result validates the docking results which also showed higher docking for hemolin-TLR4-MD2 complex than TLR3 complex.

### Codon optimization and *in silico* cloning of hemolin

Codon optimization was carried out using JCAT server to analyze the expression and stability of hemolin in *E. coli K12* expression system. The hemolin amino acid sequence was reverse translated into cDNA for codon optimization and GC content analysis. The codon adaptation was found to be 1.0 which is optimal for prokaryotic expression system. The GC content was observed to be 52.6%, which lies within the optimal range (30–70%) and is favorable for stable expression of hemolin in *E. coli K12* system. The hemolin gene was cloned into pET 30a vector by the addition of Not1 and Xho1 restriction sites, using Snapgene standalone software. The final construct was observed to be 6791 bp (Supplementary Fig. 2).

## Conclusion

Vaccines usage has become mandatory to stay from infectious diseases. Though, designing of vaccines still pose a greater challenge for vaccine development. Predominantly, the outer membrane proteins or coat proteins have high antigenic properties but they lack immunogenicity. This disadvantage can be sorted out by attaching a highly antigenic carrier protein with the non-immunogenic antigen to increase its immunogenicity. In this study we have identified a silkworm immune protein hemolin, which is highly immunogenic and has suitable properties to be viable carrier protein for vaccine production. CTL and HTL epitope prediction revealed highly antigenic surface epitopes which were capable of mounting strong adaptive immune response. B-cell epitope prediction exposed the presence of liner and discontinuous epitope in the hemolin protein, thereby facilitating a long lasting memory and herd immunity. IFN-γ epitope and antigenicity prediction illustrated the high antigenic nature of hemolin. Allergenicity and physiochemical properties of hemolin were found to be very satisfactory for carrier protein development. The secondary and tertiary structure prediction of hemolin gave a clear view of the structural properties. Molecular docking and dynamics with TLR3 and TLR4-MD2 validated the strong interaction and stability of hemolin with TLRs. The strong interaction with TLRs facilitates innate immune response, maturation of naïve B cells and adaptive T cell response. The codon optimization also gave positive CAI score, which will be helpful for *in-vivo* expression studies in near future. In our study we made use of various immunoinformatics tools for analyzing different properties of hemolin. The combined physiochemical and immunogenic properties of hemolin tends to make it a suitable candidate as carrier protein in vaccine development.

## Methodology

### Hemolin protein sequence search and collection

The hemolin gene (Uniprot ID: C4PAW6) from silkworm, *Bombyx mori* was retrieved in FASTA format from UniProt (Universal Protein Resource) database (http://www.uniprot.org/uniprot). Tertiary structure of human TLR3 (PDBID: 1ZIW) and TLR4 MD2 complex (PDBID: 3FXI) were retrieved from protein data bank (www.rcsb.org/pdb/home/home.do).

### Helper T Lymphocytes (HTL) epitope prediction

The HTL epitopes of 15-mer length for hemolin protein was predicted by IEDB (http://tools.iedb.org/mhcii/) server^24^. All epitopes were predicted for mouse MHC class II alleles (IAb, IAd, IAs, IEb, IEd and IEs) based on IC_50_ values and percentile rank; A peptide with highest affinity have IC_50_ values < 50 nM, whereas peptides with intermediate affinity have IC_50_ values < 500 nM while peptides having IC_50_ value < 5000 nM shows the least affinity, therefore least IC_50_ value shows highest affinity. Along with IC_50_ value of peptides, a percentile rank is developed by comparing the IC_50_ values of peptides against a set of random antigen from SWISSPROT database, compounds with least percentile rank show high affinity.

### Cytotoxic T Lymphocytes (CTL) epitope prediction

Cytotoxic T lymphocyte (CTL) epitopes prediction is essential for subunit vaccine development. NetCTL 1.2^25^ was utilized to predict the CTL epitopes for hemolin protein based on MHC class-I binding affinity, TAP transport efficiency and proteasomal C-terminal cleavage. Scores of all three predictions were merged together and sensitivity or specificity values can be achieved by the conversion of the threshold from the merged score, whereas 0.75 was set as a threshold value for CTL epitope identification.

### B-cell Epitope predictions

BCPRED: B Cell Epitope Prediction Server (http://ailab.ist.psu.edu/bcpred/predict.html) was used for the prediction of linear B-cell epitopes for hemolin protein achieved. BCPRED epitope prediction is based on three methods (i) AAP method (ii) BCPred and (iii) FBCpred. AAP approach is based on the finding that particular amino acid pairs occur more frequently in epitope than nonepitope sequence^26^. BCPred method employs subsequence kernel-based SVM classifier. The performance of BCPred (AUC 0.758) outperforms implementation of AAP (AUC 0.7). The cutoff score of BCPreds is >0.8 for prediction of linear B-cell epitopes FBCPred is a novel method developed for prediction of B-cell epitopes with flexible length. B-cell discontinuous epitopes prediction of the hemolin was done by ElliPro (http://tools.iedb.org/ellipro/)^27^. ElliPro provides the score to each output epitope which described as PI (Protrusion Index) value averaged over each epitope residue. For each epitope residues, the PI value is calculated on the basis of the center of mass of residue residing outside the largest possible ellipsoid. A number of ellipsoids approximated the tertiary structure of protein. The PI value of residues is based on R (a distance between two residue’s center of mass, in Å). More prominent the value of R, more will be the number of discontinuous epitopes predicted.

### IFN-γ inducing epitope prediction

Interferon gamma (IFN-γ) plays an important role in adaptive and innate immune response by stimulating macrophages and natural killer cells and provides a heightened response of MHC antigens. IFN-γ epitopes were predicted from IFNepitope server (http://crdd.osdd.net/raghava/ifnepitope/scan.php)^28^. The prediction was performed by motif and support vector machine (SVM) hybrid approach. The server is based on a dataset which consist of IFN-γ inducing and non-inducing MHC class-II binder, which can activate T-helper cells.

### Allergenicity and antigenicity prediction

Allerdictor fast allergen prediction tool (http://allerdictor.vbi.vt.edu/) was used to check the allergenicity of the protein. The allergens and non-allergens were identified using a classic SVM method^29^. Antigenicity of hemolin protein was predicted by online server ANTIGENpro (http://www.scratch.proteomics.ics.uci.edu/), to check whether the protein is antigenic or not. The server is based on the input sequence, free from any alignment and does not depend on any pathogen identity for the prediction of antigenicity. Prediction is a two-step process which is based on five algorithms and multiple representations of the sequence^30^. A summarize result of prediction was generated by SVM classifier which tells about the probability of a peptide carrying characteristics of antigen. For further conformation, hemolin protein was analyzed with VaxiJen v2.0 (http://www.ddg-pharmfac.net/vaxijen/VaxiJen/VaxiJen.html) antigen prediction server. For highest accuracy, a threshold value of 0.5 was used to check the antigenicity of each full length protein^31^.

### Prediction of various physicochemical properties

The physicochemical properties of hemolin were determined by using ProtParam tool (http://web.expasy.org/protparam/)^32^. It computes various physicochemical properties such as molecular weight, half-life, sequence length, aliphatic index, instability index, theoretical pI and grand average of hydropathicityon the basis of primary amino acid sequence.

### Secondary and Tertiary structure prediction

Secondary structure of hemolin protein was predicted by PSIPRED server (http://bioinf.cs.ucl.ac.uk/index.php?id=779) and it determines the percentage of helix, stands and coils^33^. An average Q_3_ score of PSIPRED 3.2 server is 81.6%. The tertiary structure of hemolin protein was predicted by web based server RaptorX (http://raptorx.uchicago.edu/StructurePrediction/predict/) based on template available.RaptorX generates high-quality tertiary structure models by multiple templates, the confidence score of the prediction gives an idea about the quality of predicted model. Confidence scores consist P-value and GDT (global distance test) for the relative global quality and absolute global quality respectively^34^.

### Tertiary structure refinement and validation

The predicted tertiary structure was refined by GalaxyRefine server (http://galaxy.seoklab.org/cgi-bin/submit.cgi?type=REFINE). Refinement was achieved by subsequent overall relaxation and repeated structural perturbation by molecular dynamics simulation^35^. The refinement method used by the GalaxyRefine server has been successfully tested. Further, validation of tertiary structure of hemolin protein was done by RAMPAGE (http://mordred.bioc.cam.ac.uk/~rapper/rampage.php). RAMPAGE result includes the percentage of residues in allowed and disallowed regions which define the quality of modeled structure. The ProsaWeb server (https://prosa.services.came.sbg.ac.at/prosa.php)^36^ calculates the overall quality of the tertiary structure of the protein and ERRAT (http://services.mbi.ucla.edu/ERRAT/)^37^ calculates the total non-bonded interaction and scores the protein based on the free bonds.

### Immune cell receptor-hemolin interaction investigation by molecular docking

Molecular docking was performed to check the interaction between hemolin protein and TLR-3 and TLR4-myeloid differentiation factor 4 (MD4) using PatchDock (http://bioinfo3d.cs.tau.ac.il/PatchDock/) server^38^. Patchdock is a fully automated protein - protein docking online server. Algorithm of PatchDock is divided into three different stages; the first one is molecular shape representation, followed by filtering and scoring. Firedock server was used to refine the docked complexes based on the least global score.

### Molecular dynamics simulation

Molecular dynamics (MD) simulation was used to study the structural stability of the protein. To understand the structural properties and interaction between hemolin, TLR-3 and TLR4 MD4 at the microscopic level, molecular dynamics simulation study was performed by using Gromacsv5.1.4^39^. GROMOS96 43A1 force field and the particle mesh Ewald summation method was used to run full system MD simulation by Gromacs. Energy minimization was performed prior to simulation to ensure that the geometry of the system is appropriate and there are no steric clashes by the use of steepest descent algorithm approach. System equilibration was performed in a two-step process, the first step is NVT while another step is NPT ensemble, both steps uses leap frog algorithm. In the system, equilibration steps the temperature was raised up to 300 K and pressure up to 1 bar. After completion of system equilibration, a 20 ns molecular dynamics simulation was attained for trajectory analysis. Data analysis was done using GROMACS in-built analysis tools; free energy surface plot, free energy of binding and protein ligand interaction network based on the free energy contributions were estimated.

### Codon optimization and *In silico* cloning

Reverse translation and codon optimization were performed using JCAT (Java Codon Adaptation tool) server (http://www.jcat.de/) for cloning and its expression of hemolin gene in the proper vector^40^. JCAT provides an output as cDNA sequence, which is further analyzed for codon optimization, codon adaptive index (CAI) and GC content. Lastly, NotI and XhoI restriction site was added to the cDNA sequence of hemolin gene and cloned into pET 30a vector using Snapgene standalone software.

## Electronic supplementary material

Supplementary information

## References

[1] Knuf M, Kowalzik F, Kieninger D (2011). Comparative effects of carrier proteins on vaccine-induced immune response. Vaccine.

[2] MacDonald NE (1998). Induction of immunologic memory by conjugated vs plain meningococcal C polysaccharide vaccine in toddlers: a randomized controlled trial. JAMA.

[3] Dagan R, Poolman J, Siegrist C (2010). Glycoconjugate vaccines and immune interference: A review. Vaccine.

[4] Lindstrom-Dinnetz I, Sun SC, Faye I (1995). Structure and expression of Hemolin, an insect member of the immunoglobulin gene superfamily. Eur. J. Biochem..

[5] Lanz-Mendoza H, Bettencourt R, Fabbri M, Faye I (1996). Regulation of the insect immune response: the effect of hemolin on cellular immune mechanisms. Cell. Immunol..

[6] Hoffmann Ja (1995). Innate immunity of insects. Curr. Opin. Immunol..

[7] Kannan M (2016). Proteomic analysis of the silkworm midgut during larval-pupal transition. Invertebr. Surviv. Journall.

[8] Lindström-Dinnetz I, Sun S-C, Faye I (1995). Structure and Expression of Hemolin, an Insect Member of the Immunoglobulin Gene Superfamily. Eur. J. Biochem..

[9] Lee WJ, Lee JD, Kravchenko VV, Ulevitch RJ, Brey PT (1996). Purification and molecular cloning of an inducible gram-negative bacteria-binding protein from the silkworm, Bombyxmori. Proc. Natl. Acad. Sci. USA.

[10] Shaik HA, Sehnal F (2009). Hemolin expression in the silk glands of Galleria mellonella in response to bacterial challenge and prior to cell disintegration. J. Insect Physiol..

[11] Hirai M, Terenius O, Li W, Faye I (2004). Baculovirus and dsRNA induce Hemolin, but no antibacterial activity, in Antheraeapernyi. Insect Mol. Biol..

[12] Bettencourt R, Lanz-Mendoza H, Lindquist KR, Faye I (1997). Cell adhesion properties of hemolin, an insect immune protein in the Ig superfamily. Eur. J. Biochem..

[13] Daffre S, Faye I (1997). Lipopolysaccharide interaction with hemolin, an insect member of the Ig-superfamily. FEBS Lett..

[14] Yu XQ, Kanost MR (2002). Binding of hemolin to bacterial lipopolysaccharide and lipoteichoic acid. An immunoglobulin superfamily member from insects as a pattern-recognition receptor. Eur J Biochem..

[15] O’Neill LAJ, Golenbock D, Bowie AG (2013). The history of Toll-like receptors-redefining innate immunity. Nat. Rev. Immunol..

[16] Akira S, Takeda K (2004). Toll-like receptor signalling. Nat. Rev. Immunol..

[17] Viriyakosol S, Tobias PS, Kitchens RL, Kirkland TN (2001). MD-2 Binds to Bacterial Lipopolysaccharide. J. Biol. Chem..

[18] Perales-Linares R, Navas-Martin S (2013). Toll-like receptor 3 in viral pathogenesis: Friend or foe?. Immunology.

[19] Lee K-G (2012). Bruton’s tyrosine kinase phosphorylates Toll-like receptor 3 to initiate antiviral response. Proc. Natl. Acad. Sci..

[20] Ali M (2017). Exploring dengue genome to construct a multi-epitope based subunit vaccine by utilizing immunoinformatics approach to battle against dengue infection. Sci. Rep..

[21] Khatoon N, Pandey RK, Prajapati VK (2017). Exploring Leishmania secretory proteins to design B and T cell multi-epitope subunit vaccine using immunoinformatics approach. Sci. Rep..

[22] Vos Q, Lees A, Wu ZQ, Snapper CM, Mond JJ (2000). B-cell activation by T-cell-independent type 2 antigens as an integral part of the humoral immune response to pathogenic microorganisms. Immunol. Rev..

[23] Morrison DC, Ulevitch RJ (1978). The effects of bacterial endotoxins on host mediation systems. A review. Am. J. Pathol..

[24] Sidney, J. *et al*. Quantitative peptide binding motifs for 19 human and mouse MHC class i molecules derived using positional scanning combinatorial peptide libraries. *Immunome Res*. **4** (2008). 10.1186/1745-7580-4-2 PMC2248166 18221540

[25] Larsen MV (2007). Large-scale validation of methods for cytotoxic T-lymphocyte epitope prediction. BMC Bioinformatics.

[26] El-Manzalawy Y, Dobbs D, Honavar V (2008). Predicting linear B-cell epitopes using string kernels. J. Mol. Recognit..

[27] Ponomarenko J (2008). ElliPro: a new structure-based tool for the prediction of antibody epitopes. BMC Bioinformatics.

[28] Dhanda SK, Vir P, Raghava GP (2013). Designing of interferon-gamma inducing MHC class-II binders. Biol. Direct.

[29] Dang HX, Lawrence CB (2014). Allerdictor: fast allergen prediction using text classification techniques. Bioinformatics.

[30] Magnan CN (2010). High-throughput prediction of protein antigenicity using protein microarray data. Bioinformatics.

[31] Doytchinova IA, Flower DR (2007). VaxiJen: a server for prediction of protective antigens, tumour antigens and subunit vaccines. BMC Bioinformatics.

[32] Gasteiger, E. *et al*. Protein Identification and Analysis Tools on the ExPASy Server. In *The Proteomics Protocols Handbook* 571–607, 10.1385/1592598900 (2005).

[33] McGuffin LJ, Bryson K, Jones DT (2000). The PSIPRED protein structure prediction server. Bioinformatics.

[34] Källberg M (2012). Template-based protein structure modeling using the RaptorX web server. Nat. Protoc..

[35] Sellers BD, Zhu K, Zhao S, Friesner RA, Jacobson MP (2008). Toward better refinement of comparative models: Predicting loops in inexact environments. Proteins Struct. Funct. Genet..

[36] Wiederstein, M. & Sippl, M. J. ProSA-web: Interactive web service for the recognition of errors in three-dimensional structures of proteins. *Nucleic Acids Res*. **35** (2007). 10.1093/nar/gkm290 PMC1933241 17517781

[37] Colovos C, Yeates TO (1993). Verification of protein structures: Patterns of nonbonded atomic interactions. Protein Sci..

[38] Schneidman-Duhovny, D., Inbar, Y., Nussinov, R. & Wolfson, H. J. PatchDock and SymmDock: Servers for rigid and symmetric docking. *Nucleic Acids Res*. **33** (2005). 10.1093/nar/gki481 PMC1160241 15980490

[39] Abraham MJ (2015). Gromacs: High performance molecular simulations through multi-level parallelism from laptops to supercomputers. SoftwareX.

[40] Grote, A. *et al*. JCat: A novel tool to adapt codon usage of a target gene to its potential expression host. *Nucleic Acids Res*. **33** (2005). 10.1093/nar/gki376 PMC1160137 15980527

