# Supplementary material for: Investigation of immunogenic properties of Hemolin from silkworm, Bombyx mori as carrier protein: an immunoinformatic approach
Source: Sci Rep. 2018 May 3;8:6957. doi: 10.1038/s41598-018-25374-z (PMC5934409; doi:10.1038/s41598-018-25374-z)
Supplement: Supplementary file 1 — Supplementary information [file 41598_2018_25374_MOESM1_ESM.pdf]

Investigation of immunogenic properties of Hemolin from silkworm, *Bombyx mori* as carrier protein: an immunoinformatic approach

Veeranarayanan Surya Aathmanathan[1], Nattarsingam Jothi[1], Vijay Kumar Prajapati[2], Muthukalingan Krishnan[1, 2]*

[1]Department of Environmental Biotechnology, Bharathidasan University, Tiruchirappalli, Tamil Nadu, India

[2]Department of Biochemistry, School of Life Sciences, Central University of Rajasthan, Bandarsindri, Kishangarh, Ajmer 305817, Rajasthan India

Corresponding author*
Prof. Muthukalingan Krishnan
Dean, Academic Sciences
Department of Biochemistry
Central University of Rajasthan
NH-8, Bandarsindri, Ajmer
Rajasthan Pin- 305817 India
Phone: +91 9443998251
Email: profmkrish@curaj.ac.in

Supplementary Fig 1: SMART analysis of hemolin protein

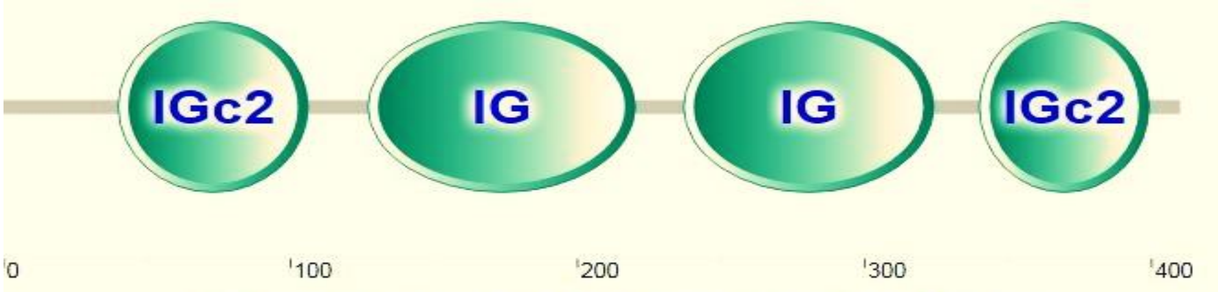

Confidently predicted domains, repeats, motifs and features:

Name ▲	Start	End	E-value
IGc2	40	106	0.000234
IG	127	220	0.000813
IG	237	324	0.000813
IGc2	340	399	4.48e-15

Supplementary Fig 2: *In silico* restriction cloning of hemolin into pET 30a expression vector

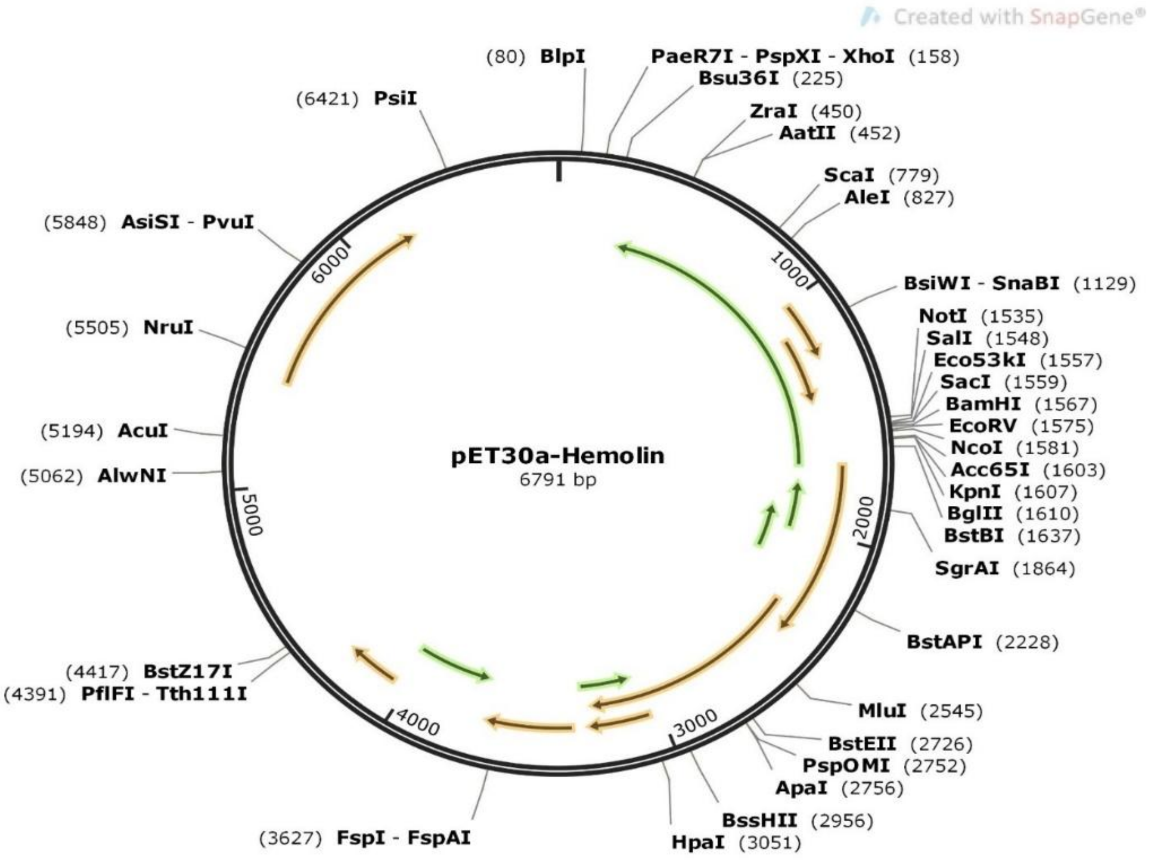

Supplementary Table 1: CTL epitope prediction by NETCTL Server

Allele	Residue Number	Sequence	Combined Score
A1 Super type	394	ASNEHGAEY	2.3802
	355	PSPLVSWTY	2.1822
	243	KVGDLTYLY	1.3822
	78	TIDAGSLVF	1.3159
	381	AVNKSNQGY	1.2778
	236	LSDHVVAKV	1.2719
	220	VTPASEPTY	1.1438
	175	YSPEGTLYF	1.1432
	174	TYSPEGTLY	1.1037
	246	DLTYLYCIY	1.0722
A2 Super type	315	LQHTITFSV	1.2590
	180	TLYFSNASL	1.1251
	212	IVTYYITQV	1.0460
A3 Super type	324	VSAPTFTTK	1.3746
	243	KVGDLTYLY	1.3168
	106	GVASTRATK	1.2626
	376	GLVIKAVNK	1.2024
	258	PLAHPSWSK	1.1563
	268	GVNVDNTYK	1.0597
	82	GSLVFTQTK	1.0508
	235	YLSDHVVAK	1.0433
	381	AVNKSNQGY	1.0121
B7 Super type	136	KPFELRCPV	1.5803
	332	KPEKRTLAT	1.5769
	148	YPKPTISWM	1.2667
	350	KATGIPSPL	1.2032
	107	VASTRATKL	1.0907
	27	VPVLKEAPA	1.0373
B8 Super type	148	YPKPTISWM	1.4365
	112	ATKLRRTYI	1.1876
	332	KPEKRTLAT	1.1646
B27 Super type	313	RRLQHTITF	2.1110
	288	RRLVIKEVW	1.3667
	39	FREGQATRL	1.3036
	116	RRTYIETPA	1.2259
	280	TRHNRSSGR	1.0800

B8 Super type	39	FREGQATRL	1.9750
	180	TLYFSNASL	1.2814
	68	HFSVGQDTL	1.1554
B44 Super type	31	KEAPAEVLF	1.4364
	160	DEDGSTENF	1.0707
	224	SEPTYGELI	1.0481
	297	AEDAGTYTC	1.0416
	369	TEGVTGDGL	1.0171
B58 Super type	321	FSVVSAPTF	1.9010
	117	RTYIETPAF	1.8816
	256	GTPLAHPSW	1.1716
	284	RSSGRRLVI	1.1498
	288	RRLVIKEVW	1.1204
	175	YSPEGTLYF	1.1119
B62 Super type	117	RTYIETPAF	1.3973
	394	ASNEHGAEY	1.3575
	175	YSPEGTLYF	1.3505
	241	VAKVGDLTY	1.2977
	381	AVNKSNQGY	1.2696
	7	LVLGTCVIY	1.2535
	130	VTVVEGKPF	1.2199
	321	FSVVSAPTF	1.2058
	97	YQCFAKSDF	1.1330
	243	KVGDLTYLY	1.0349

Supplementary Table 2: Ellipro prediction scores of linear epitope

S.No	Residue Number	Residue Name	Score
1	1	MET	0.985
2	2	ASN	0.985
3	3	SER	0.985
4	4	TRP	0.985
5	5	THR	0.985
6	6	LEU	0.985
7	7	LEU	0.983

8	8	VAL	0.978
9	9	LEU	0.976
10	10	GLY	0.963
11	11	THR	0.956
12	12	CYS	0.973
13	13	VAL	0.956
14	14	ILE	0.963
15	15	TYR	0.956
16	16	THR	0.971
17	17	THR	0.980
18	18	GLY	0.963
19	19	GLN	0.954
20	20	PRO	0.951
21	21	VAL	0.949
22	22	ASN	0.932
23	23	SER	0.880
24	24	GLY	0.795
25	25	ASP	0.688
26	26	LYS	0.471
